# Examining Burnout Among Intern Physicians During the COVID-19 Pandemic: Insights and Solutions from Qualitative Research

**DOI:** 10.3390/healthcare13030335

**Published:** 2025-02-06

**Authors:** Vithawat Surawattanasakul, Wuttipat Kiratipaisarl, Vitchayut Phetsayanavin, Chantarateera Pholvivat, Natcha Auernaruemonsuk, Chanon Lamlert, Warisa Soonthornvinit, Lakkana Hengboriboon, Penprapa Siviroj

**Affiliations:** 1 Department of Community Medicine, Faculty of Medicine, Chiang Mai University, Chiang Mai 50200, Thailand; vithawat.surawat@cmu.ac.th (V.S.); wuttipat.k@cmu.ac.th (W.K.); 2 Environmental and Occupational Medicine Excellence Center, Faculty of Medicine, Chiang Mai University, Chiang Mai 50200, Thailand; 3 Department of Psychiatry, Faculty of Medicine, Chulalongkorn University, Bangkok 10332, Thailand; victorvertick@gmail.com; 4 Department of Community, Family and Occupational Medicine, Faculty of Medicine, Khon Kaen University, Khon Kaen 40002, Thailand; chantph@kku.ac.th; 5 Department of Psychiatry, Faculty of Medicine, Ramathibodi Hospital, Mahidol University, Bangkok 10400, Thailand; natcha.aue@student.mahidol.ac.th; 6 Department of Surgery, Ratchaphiphat Hospital, Bangkok 10160, Thailand; chaaanooon2@hotmail.com; 7 Department of Community Medicine, Faculty of Medicine, Ramathibodi Hospital, Mahidol University, Bangkok 10400, Thailand; warisa.soo@mahidol.edu; 8 International College, Khon Kaen University, Khon Kaen 40002, Thailand; lakkhe@kku.ac.th

**Keywords:** intern physicians, burnout, workplace conditions, clinical practices, hospital management, COVID-19 pandemic, Thailand

## Abstract

**Background/Objectives:** The extensive exposure of physicians to the COVID-19 pandemic has contributed to occupational stress and burnout in their daily lives. This study aimed to explore the lived experiences of intern physicians who experienced burnout during the COVID-19 pandemic and to identify potential solutions to enhance clinical practices in future pandemics. **Methods:** This study employed a qualitative, phenomenological study utilizing in-depth interviews. The participants were 19 first-year intern physicians from public hospitals in Thailand, selected through a purposeful sampling approach who had experienced burnout. Semi-structured interviews were conducted face-to-face and via online platforms. A thematic narrative analysis approach was used. **Results:** Phenomenological explorations included two parts: the first explored physicians’ workplace conditions while providing patient care, and the second focused on their proposed solutions for policy changes in clinical practices and hospital management. Four main themes in the first part were derived: (1) emotional suffering and burnout; (2) engaging with a high-intensity workplace; (3) hostile work environments; and (4) deterioration of relationships with staff and colleagues. The second part identified three main themes: (1) changes in policy of clinical practices; (2) effective hospital management; and (3) building interpersonal skills. **Conclusions:** The COVID-19 pandemic has exacerbated challenges faced by intern physicians, such as high-pressure working conditions, deteriorated relationships with colleagues, and ineffective management, all of which contribute to burnout. These challenges require targeted policy changes in clinical practices, effective hospital management, and building interpersonal skills. Recommendations include improved clinical practices, increased academic support, comprehensive orientation programs, effective communication, teamwork assistance, stress management, and transforming organizational culture to value physicians during internships.

## 1. Introduction

During the COVID-19 pandemic, physicians worldwide faced exceptionally stressful working conditions, with overworked medical staff, shortages of essential supplies such as personal protective equipment (PPE) and ventilators [1,2,3], and repurposing of hospital wards and equipment to accommodate COVID-19 patients [4,5,6]. These circumstances necessitated not only the prioritization of patients and establishment of an order of medical care but also the implementation of resource rationing. This process involved the allocation of limited resources to some patients while denying them to others. Consequently, medico-legal disputes emerged, particularly when questions of accountability, negligence, or liability arose in cases involving healthcare-related COVID-19 infections affecting patients or healthcare workers [7]. Before the COVID-19 pandemic, work-related stress was inevitable in every aspect of the medical profession and everyday life. When stress becomes chronic and unresolved, more problems are likely to arise. The American Psychological Association [8] suggested that prolonged work-related stress can lead to poor health including mental and physical symptoms such as anxiety, depression, and sleep problems. The added stressors and challenges fueled by the COVID-19 pandemic conferred a significant increase in the risk of burnout for physicians, particularly intern physicians. These circumstances led to quality of life and mental health issues, such as burnout [9,10,11,12,13,14].

Burnout is a phenomenon of emotional and psychological stress characterized by feelings of cynicism and detachment. Burnout, as defined by Maslach et al., is measured by assessing three dimensions: (1) feelings of energy depletion or emotional exhaustion (EE), (2) feelings of negativism or cynicism related to one’s job or depersonalization (DP), and (3) a sense of ineffectiveness and lack of personal accomplishment (PA) [15]. Burnout has been described as a syndrome resulting from chronic workplace stress that has not been successfully managed, as defined by the International Classification of Diseases 11th Revision (ICD-11) [16]. It can have a profound impact on a physician’s well-being, affecting their ability to provide high-quality care and potentially leading to high physician turnover [17,18,19].

The medical education system in Thailand offers a six-year study period for high school diploma holders, with financial support for tuition at government institutions. Scholarship recipients serve three years after graduation, including a dedicated internship year in a provincial hospital. The first year of the internship involves rotation through diverse wards such as internal medicine, surgery, and orthopaedics and elective subjects including paediatrics, obstetrics, gynaecology, and emergency, resulting in a comprehensive twelve-month training program. Additionally, the Thai Medical Council has set specific requirements for intern physicians during their first year of training. These include a maximum of 15 duty days per month, including shifts in inpatient wards and emergency departments [20,21] and emphasizing basic procedures such as intubation and resuscitation [22]. Previous studies have shown increased stress and burnout in first-year physicians and trainees during the COVID-19 pandemic [23,24] due to a lack of support and unresponsive hospital management, resulting in increased workloads as they managed COVID-19 patients alongside their regular responsibilities. These challenges disrupted their work–life balance, affected their care quality and motivation, and were linked to burnout [25]. Moreover, physicians working in particularly challenging environments, such as conflict-affected regions of southern Thailand, experienced heightened levels of stress and burnout [26].

Recently, quantitative burnout studies have focused on assessing burnout and contributory factors, primarily prolonged exposure to work-related problems without specifying the nature of work or specific triggers [27]. Despite the pandemic being unavoidable, it created new and challenging levels of work-related stress, emotional demands, and unmanageable workloads for physicians. Stress and burnout in relation to the pandemic have become major organizational problems and necessitate immediate action to address issues pertinent to physicians’ practices, provide academic and mental health support, and enhance learning success [28]. While workplace stress and physician burnout are not new concerns, it is imperative to fill the research gap to fully understand the phenomenon of burnout through physicians’ lived experiences during the COVID-19 pandemic. It is also of great importance to explore potential solutions to prevent burnout for future pandemics. Qualitative methods are relevant in this context, as is phenomenological study to uncover the essence of the phenomenon, providing a deep, contextualized understanding that can inform practices and policy [29]. There is a particular lack of literature on intern physician burnout in the Thai context. This study comprises qualitative research aimed at exploring the lived experiences of intern physicians who experienced burnout while providing care to patients during the second and third waves of the COVID-19 pandemic, which had higher infection rates than the initial wave in Thailand [30], as well as identifying potential solutions to enhance clinical practices and mitigate burnout in critical and high-pressure situations.

## 2. Materials and Methods

### 2.1. Study Design

This qualitative research employed a descriptive phenomenological approach to explore the lived experiences of intern physicians who completed their first year of internships in governmental hospitals and experienced burnout during the COVID-19 pandemic from 2021 to 2022 in Thailand. The phenomenological approach aims to gain an in-depth understanding of individuals’ everyday lived experiences or phenomenon and distil them into their central meaning or essence [29,31]. In this approach, the unit of analysis comprises individuals who have encountered similar experiences of the phenomenon under investigation. Data collection primarily relies on in-depth interviews, which facilitate a comprehensive exploration of participants’ perspectives. Descriptive phenomenology is particularly well-suited for studies seeking to capture and articulate the essence of lived experiences [32,33]. By analyzing and comparing participants’ narratives, researchers can identify commonalities and define the core elements of the phenomenon. This method can generate new meanings for such experiences [34]. The experience of living and working with the COVID-19 pandemic represents a novel and unprecedented challenge for healthcare professionals. Therefore, a descriptive phenomenological design was deemed appropriate to investigate the unique experiences of intern physicians during this period and enabled an in-depth exploration of their perspectives, capturing the realities of their professional lives as shaped by the pandemic.

The processes of this design began with the identification of specific cases from a previous cross-sectional study [35], which surveyed 241 intern physicians in Thailand who had directly experienced the phenomenon of interest, burnout symptoms, as determined through a web-based online survey. Data collection primarily involved in-depth, semi-structured interviews, allowing participants to share their experiences in their own words. Additionally, reflective journals were utilized to further capture the essence of their lived experiences. The analysis process followed a systematic approach which included coding significant statements, clustering themes, and developing a comprehensive description of the phenomenon experienced.

### 2.2. Participants

The participants in this study were selected using purposive sampling. A total of 45 intern physicians, who had previously responded to our study [35], voluntarily agreed to participate. Inclusion criteria required participants to have graduated from a six-year medical school program in Thailand and to be in their first year of internships at a government hospital. Exclusion criteria included physicians who did not experience burnout during their internships, as indicated by Maslach Burnout Inventory (MBI) scores, or if they self-reported a diagnosed mental health condition. Participants were allowed to withdraw from the study at any point during the interviews. Based on these criteria, 20 participants who did not meet the burnout criteria were excluded, leaving 25 eligible for interviews. Of these, six participants were unavailable during the interview period. Finally, 19 intern physicians, representing all regions of Thailand, including both genders and various hospital affiliations, were interviewed. While this sample provided sufficient data to achieve the study objectives, its size may limit the generalizability of the findings.

Table 1 provides an overview of the general characteristics of the participants. Among the 19 participants, 6 were male and 13 were female, with ages ranging from 24 to 26 years. All participants were single, had completed a six-year medical school program, and had less than one year of work experience. Most participants (14 out of 19) worked at Ministry of Public Health (MOPH)-affiliated hospitals, with 47.4% working in hospitals with less than 500 beds. It is worth noting that only four hospitals offered teaching for resident physician training.

### 2.3. Data Collection

We conducted face-to-face semi-structure interviews via Zoom meetings online, utilizing open-ended questions, between 21 June and 27 July 2022. Data were gathered at the participants’ preferred times. Participants provided verbal informed consent through web conferences and agreed to have their interviews recorded. The interview began with initial questions designed to encourage participants to share information about their job description and clinical practice rotation during their first year of work. These interview questions encompassed various dimensions of experiences in their workplace based on the participants’ responses, and the researcher asked detailed follow-up questions to delve deeper into their experiences (Table 2). For the pilot test to establish the open-ended questions, we interviewed two intern physicians who did not participate in the main study. The pilot findings led to adjustments to the final interview process, including reducing ambiguity, incorporating additional probing questions, and adjusting the interview guidelines to ensure a more natural flow of conversation and participant comfort. All interview sessions were recorded and subsequently anonymized, with all private details removed before transcription. Additionally, we conducted a single round of repeated interviews with three participants to refine the questions and ensure clarity.

After 19 in-depth interviews, saturation was reached, as no new information or insights emerged from subsequent interviews. This implied that the researcher “has the story” and additional participants would only repeat it. At this point, the researcher had enough data to capture the breadth and depth of the phenomenon, enabling the construction of category attributes. Following saturation, the researchers translated the collected data from Thai to analyze it. These data, consisting of notes and audio recordings, were transcribed and compiled into a Microsoft Word document for further analysis. The translation process involved an initial translation from Thai to English conducted by two researchers proficient in both languages. This was followed by a review conducted by native English speakers to ensure the accuracy and clarity of the translated content. Throughout the process, researchers collaborated closely with native English speakers to preserve culturally specific expressions and meanings, ensuring that the essence of participants’ experiences was accurately conveyed.

### 2.4. Data Analysis

The qualitative data were analyzed and processed using the Colaizzi 7-step technique that ensures the credibility and reliability of its results [36,37], including direct quotations. The seven steps are as follows: (1) reading and rereading all the transcripts; (2) extracting significant statements that pertain to the phenomenon; (3) formulating meanings from the significant statements; (4) creating theme clusters and subthemes; (5) an exhaustive description of the phenomenon; (6) a description of the fundamental structure of the phenomenon; and (7) participant validation.

We transcribed and re-examined the interviews to ensure the data were error-free. The research team thoroughly reviewed and validated each participant’s transcription, addressing any unclear elements and questions. Data were interpreted and coded. A thematic analysis approach was used. After analyzing the data, themes and subthemes were extracted. Extracted themes were returned to the participants for verification. To explicate themes, significant statements with similar terms and their formulated meanings were grouped to form theme clusters, and related theme clusters were then aggregated together to establish themes. We removed redundant or misused descriptions of the phenomenon. Finally, the findings were sent to the participants, who considered the findings and verified that they depicted their experiences of the phenomenon accurately.

### 2.5. Rigor and Trustworthiness

The Guba and Lincoln criteria for assessing the trustworthiness of findings were used in this study [38]. We employed recording equipment and transcribed digital files to improve the dependability of comprehensive field data. Peer debriefing was utilized to share and discuss research findings and interpretations with experienced colleagues such as staff and hospital managers to enhance credibility. For transferability, the data collection process, analysis process, and results were briefly conveyed to the hospital manager and supervisor as having the overall responsibility of intern physicians to ensure a general comprehension of the statements. This was done to validate the reliability and applicability of the findings. Additionally, an overview of the research findings, along with quotations, was sent to three participants to confirm their experiences were accurately recorded. It was verified that the investigator had accurately recorded their experiences. The researcher also validated their results by presenting the data to three researchers who met the inclusion criteria but did not participate in this study.

### 2.6. Ethical Considerations

Ethical approval for this study was provided by the Institutional Review Board at the Faculty of Medicine, Chiang Mai University (approval no. 079/2022; date of approval: 24 February 2022). Verbal informed consent was obtained by the first author, including approval to use verbatim quotes from interviews, while clear communication that participants would be personally identifiable in the research reports. The participants’ right to withdraw was detailed and discussed with them throughout the study via both email and online meetings. Before finalizing the research, participants were given the opportunity to review the analysis of their case and either fully withdraw from the study or agree on a preferred level of re-identification. None of the participants chose to request changes.

## 3. Results

Based on 19 interviews with two parts to the study, physicians’ experience with burnout, and suggested potential solutions, a total of six themes emerged from the 16 subthemes as listed in Table 3.

### 3.1. Experiencing Burnout During Intern Physician’s Life

#### 3.1.1. Emotional Suffering and Stress

The COVID-19 pandemic has profoundly affected the mental health of intern physicians. Increased stress and burnout due to daily responsibilities and restrictions limiting travel and social interactions outside of work have posed significant challenges. Some intern physicians reported utilizing various coping mechanisms, such as self-reflection and emotional expression, to manage stress and burnout. These strategies involve seeking support through communicating with friends and family, participating in leisure activities, engaging in exercise, and consuming calorie-dense meals.

**(a) Exhaustion and pressure from daily responsibilities.** Most intern physicians mentioned feeling anxious and extremely exhausted, a lack of motivation, dizziness, and trouble thinking, as if everything was going slowly. These have been shown to result in reduced cognitive efficiency, loss of interest in activities, and sadness triggered by specific events. One experienced an episode of crying in the outpatient department.

“*I feel anxious and unmotivated to get up and go to work. There were times when I would cry in the outpatient department, happening about 3 out of 5 days.*”

(P1)

“*It’s a station for sad people, I’m sad that both my workload and the coworkers (interns) made me feel as though my insides were dead like I was a tree being pierced by ants all the time.*”

(P5)

“*I’m feeling dizzy and struggling with thinking as if everything is moving in slow motion. I noticed decreased cognitive efficiency and fluctuating emotions…burnout made me like a tree without deep roots that are still alive but can be eliminated.*”

(P8)

**(b) Unhappy: lifestyle and imbalance in work and play.** The prolonged duration of the COVID-19 pandemic exposed most intern physicians to harmful events while caring for patients. Despite the risks, they fulfilled their responsibilities, which affected the quality of care and work–life balance.

“*Because my routine work finished late, I had to work a lot without taking a day off and traveling far from home. I occasionally had to work in the inpatient department (IPD) from 5 to 6 in the morning. My grandma called me to remind me to go home for a holiday dinner.*”

(P5)

“*I don’t derive enjoyment from my work, and at times, I blame myself. I make a conscious effort not to mistreat people I interact with…lack motivation, and reluctance to engage in activities…it’s challenging to ask people…I feel burdened with making decisions, afraid of making the wrong choice, and insecure.*”

(P16)

#### 3.1.2. Engaging in High-Intensity Workplace

Intern physicians were assigned to work with general practitioners, providing initial medical treatment across hospital departments. They were exposed to the COVID-19 pandemic during their first year of internships, which caused troubling experiences as follows:

**(a) Extreme workload.** The COVID-19 pandemic brought unprecedented challenges, particularly during its two largest waves in Thailand. Throughout this period, all physicians were reassigned to the acute respiratory infection (ARI) clinic to care for COVID-19 patients. All participants reported a significant increase in their workload. Their responsibilities included starting ward rounds early in the morning, working extra hours, and covering shifts on both Saturdays and Sundays. They typically had only one weekend and one day off each month, with no holidays, and no time off after working night shifts.

“*Because of the requirements of working at the ARI clinic, few physicians worked in other departments to treat patients, thus my main responsibility was to treat more patients.*”

(P1)

“*I had an excessive amount of work to do…work all the time…It’s unreasonable to work from when it gets dark till it gets light in the morning…sometimes 10 cases per night were admitted.*”

(P8)

“*Heavy work, a big job, working past the designated end time, not clearing the work, and being extremely busy… COVID-19 caused the hardest job.*”

(P9)

**(b) Facing complicated tasks beyond responsibilities.** First-year internships involved clinical practice in tertiary hospitals, including emergency departments, internal medicine, paediatrics, and delivery rooms. Some participants worked in complicated conditions, including challenging patients with severe symptoms. These tasks were outside their scope, not recognized, and raised pressure.

“*Intern physicians, despite being at the lowest level of the medical profession, have enormous responsibilities in dealing with severe and complex cases including cardiac arrest.*”

(P3)

“*There are several procedures that require prior guidance that are not covered by the general practitioner and can cause harm to the patients due to their difficult procedure.*”

(P17)

“*I was able to deliver triplets and a baby with a low birth weight during my extra-duty shift alone.*”

(P13)

**(c) Highly demanding but unable to control.** Intern physicians reported that nurses double-checked orders, disregarded them, and verified instructions with staff, reducing the confidence of the interns. However, the interns expressed frustration with the staff’s inconsistent treatment and procedures. Contradictory instructions reduced the control of the work of the intern physicians, slowed the process, and increased poor decision-making. This led to confusion and reduced efficiency, increasing anxiety and affecting the overall work environment.

“*Nurses double-checked that they had informed the staff, which slowed down the process even more…despite the intern physician’s ability to order, there are many patients, so keep checking back and forwards.*”

(P6)

“*I consulted some cases with staff, and he asked many questions…didn’t believe my evaluation…should be investigated and re-evaluated repeatedly.*”

(P14)

#### 3.1.3. Hostile Work Environments

Intern physicians may demonstrate lower medical professionalism and be exposed to hospital environments. They are still learning to adapt to their roles, which are in a critical phase of learning. Additionally, intern physicians faced challenges in managing relationships with staff and their clinical training skills during their first-year internship, as they worked on a rotation schedule, spending two months in each department. The pandemic exacerbated these difficulties, with instances of humiliation by colleagues further straining relationships and hindering skill development during their first-year internship.

**(a) Insufficient work support.** Some intern physicians reported the absence of effective support systems, unclear workflows, and a lack of structured psychiatric care programs for physicians—the lack of clinical guidelines created uncertainties and raised questions about appropriate investigation and treatment. Some interns reported negative experiences with staff members and nurses, with 20% exhibiting unpleasant behavior. Issues included obtaining consultations and referrals, especially when seeking phone assistance. Staff neglected mentorship responsibilities for the clinically ill, prioritizing their work over providing guidance.

“*At the same time, it is extremely hard to ask the staff for advice…it’s like being alone…there is no official support system; instead, personal connections and peer-to-peer assistance between interns serve this purpose.*”

(P3)

“*There are several procedures that must be carried out without prior guidance...no system of support, no mental health program for doctors.*”

(P17)

“*I had no staff accessible to help…the only person who provided help was a nurse...I encountered several problems obtaining advice from the referral hospital while working at the community hospitals.*”

(P4)

“*I would get anxious if I had to work with someone…he was one of those toxic people who made me feel nervous and depressed at work…criticizing interns over a small mistake.*”

(P5)

**(b) Being humiliated by colleagues.** Unprofessional behavior by staff, nurses, and patients, including public reprimands of intern physicians, abuse of power to damage their reputation, and inappropriate harassment of interns (including sexual, physical, and verbal mistreatment), has significant negative impacts.

“*The nurses genuinely hurt my feelings...both the use of bad language and the pressure to investigate the patient quickly, particularly before finishing daily work.*”

(P1)

“*There was the use of physical, verbal, and sexual abuse…touching my body…staff called me ‘a fuck...idiot’ in front of other people and said to the patient’s cousin your problems because of this doctor.*”

(P4)

“*Complaints of sexual harassment by staff have been made…the story comes from those in positions of authority, so I won’t since it will affect my ability to learn.*”

(P8)

**(c) Interns’ voices: remaining unresolved.** Hospital managers struggled to address the concerns of intern physicians that were both work- and service-related, leading to discomfort in raising or having additional conversations with managers, as some issues remained unresolved.

“*Staff who are on duty but not working with interns. The medical staff on duty did not work with the interns, and hospital administration was unable to bring the staff involved in the problem to the discussion meeting, making it impossible to find a solution*.”

(P16)

**(d) Shame-driven learning.** During their training, physicians observed a concerning practice termed shame-driven learning, where interns were publicly criticized for their mistakes, sometimes on social media or in front of large audiences, leading to significant embarrassment. This practice was particularly harsh for interns carrying out medical treatment that posed dangers or was potentially life-threatening to patients. There were notable discrepancies in how these situations were handled: while interns were criticized, staff were not blamed for similar incidents, leading to unfairness and inconsistency in accountability.

“*Some staff humiliate others by using their expertise…reviling themes via social media specifically for doctor groups that include more than 400 members just for a minor mistake.*”

(P6)

“*When staff made errors or mistreated patients, these incidents were often unreported or not addressed in academic meetings. In contrast, if an intern’s actions endangered a patient, the fault would be promptly reported…interns would face significant criticism and accountability measures.*”

(P14)

### 3.2. Suggesting Potential Solutions for Clinical Practices and Hospital Management

#### 3.2.1. Changes in Policies for Clinical Practices

Intern physicians addressed recommendations for changes in clinical practice management aimed at newly arrived intern physicians, focusing on effective workforce management, shift allocation, and embracing learning opportunities. Hospitals can create an environment that supports effective workforce management while also fostering a rich learning experience through strong workflow processes for intern physicians.

**(a) Effective workforce and shift allocation.** Hospital managers should ensure that shifts are distributed equitably among interns, allowing each intern to experience a variety of departments without overburdening any individual. Internships should focus on learning and growth, encouraging interns to proactively seek out learning opportunities. Additionally, provision of adequate rest periods is essential to promote both quality care and a good quality of life for interns.

“*Increasing manpower and fair distribution of the workload… should adjust manpower to suit the job...should be flexible and able to change.*”

(P1)

“*Organizing the inspection system, dividing people into alternating ARI/OPD, and organizing them into more steps or adding personnel so the workload is reduced, resulting in better work quality and quality of life.*”

(P2)

**(b) Strong workflow process.** Hospitals should establish workflow processes and regularly update streamline operations and make job responsibilities clear for intern physicians. Ensure consultations and learning opportunities are integrated into daily clinical practice, encouraging interns to reflect on their experiences and learn from them.

“*Workflow processes should be established and regularly updated to streamline operations, making job responsibilities clear, and establishing clear duties and responsibilities of intern physicians.*”

(P1)

“*I want an inspection and follow-up to make sure that staff members are performing their jobs, reporting for duty, and getting consultations following their agreements.*”

(P12)

#### 3.2.2. Effective Hospital Management

Intern physicians have provided recommendations for effective management aimed at supporting new intern physicians. Hospitals should promote a mutual respect culture between staff and interns, create a supportive and educational environment that fosters growth, and ensure mental support for a high-quality work life for intern physicians.

**(a) Promotion of a culture of mutual respect between staff and interns.** Promoting a culture that values honest and constructive feedback is key to the growth and success of intern physicians. Hospitals can create an environment where interns feel safe and supported in sharing their experiences. It is important to assure interns that their feedback will be taken seriously and that any concerns or suggestions they raise will be addressed promptly and thoughtfully.

“*There should be feedback on problems on a ward basis for executives to listen to the problems and join in solving them…intern physicians should be thought of as colleagues.*”

(P1)

“*Staff members should not judge interns or their thoughts and decide they can’t do something...but should teach the younger ones and not try to use derogatory words.*”

(P2)

**(b) Anonymous reporting.** Hospitals should implement an anonymous workplace reporting system that can create a safer, more supportive environment and significantly enhance the effectiveness of clinical practice program reviews for intern physicians. They should also regularly review and analyze feedback to identify areas for improvement.

“*In response to complaints and concerns, a committee should be formed, including executives, staff, nurses, and relevant stakeholders alongside interns.*”

(P4)

“*Follow-up and clarification should be routine after improvements are made, and any resolved issues should be communicated, along with plans, to interns…a system that allows us to hear the voices of actual workers on the job site is something…I would want to see it established.*”

(P7)

**(c) Strengthening mental support.** Providing on-site counselling services for intern physicians can play a vital role in supporting their mental health and overall well-being. The demanding nature of their work can lead to high levels of stress and burnout, making access to professional mental health support essential. By ensuring these services are readily available, hospitals can help interns cope with the challenges they face and ultimately enhance their professional development.

“*To implement a mental health support system for interns, addressing issues like stress and burnout, with readily available urgent treatment options.*”

(P1)

#### 3.2.3. Building Interpersonal Skills

**(a) Effective communication.** By clearly communicating the actions taken in staff response feedback to intern physicians, hospitals can foster a sense of trust and transparency. This process should involve not only addressing the issues raised but also keeping the interns informed about the steps being taken to resolve them. Staff members should actively assist in collecting feedback, helping to identify and solve issues, and ensuring that the feedback loop is closed.

“*Find someone you can trust to talk or consult with…don’t keep problems to yourself.*”

(P5)

“*Problems in work are only in communication…to better understand one another, we should work to correct and improve them.*”

(P6)

**(b) Self-development before graduation.** Intern physicians offer valuable advice on preparing new physicians for challenges. They suggest incorporating training regarding the hospital systems during the sixth year of medical education, allowing new physicians to hit the ground running when they start their internships. Additionally, they recommend managing emotional stress through mindfulness training, fostering a positive perspective, and setting realistic expectations. They also emphasize the importance of rest, self-care, self-kindness, and coping with challenges and mistakes.

“*Develop skills such as having a positive attitude and building a support network to maintain an optimistic mindset. Minimal stress accumulation while confronting reality head-on. Take a break if you’re exhausted.*”

(P6)

“*Have a complete understanding of the hospital system before they graduate…need to begin preparing for study in year 6th.*”

(P9)

“*Be kind to yourself and give yourself plenty of comfort.*”

(P16)

## 4. Discussion

This study explored the lived experiences of intern physicians providing care to patients affected by burnout during the COVID-19 pandemic, employing qualitative research based on descriptive phenomenology. The findings reveal that intern physicians faced significant suffering and chronic stress, consistent with previous studies on the challenges faced by healthcare personnel during the COVID-19 pandemic [13,23,24]. Drawing on Matthew Ratcliffe’s concepts of existential feelings, fundamental orientations that shape individuals’ perceptions and interpretations of the world, the study suggests that fear during the pandemic extended beyond a mere response to the virus, reflecting deeper disruptions in perceptions of safety and future possibilities [39,40]. Intern physicians reported working in high-intensity environments with excessive workloads and complex procedures, which did not align with the Thai Medical Council’s recommendations on work hours and mandatory breaks [41]. Additionally, significant challenges in maintaining a work–life balance were observed, reinforcing a previous finding that related imbalance with burnout in times of increased professional demands [25]. Intern physicians also reported limited autonomy in managing their professional responsibilities, particularly in the face of excessive job demands. The study also highlighted a lack of job autonomy among interns, particularly when job demands were high, a challenge that can be analyzed using the Karasek model to understand the relationship between job demands and control [42].

Intern physicians emphasized the deterioration of the relationship between staff and interns and the importance of enhancing colleague support and recognizing the collective nature of their team. However, they noted that nurses and staff often fail to follow treatment orders due to pressure to manage the entire ward, resulting in some patients being deprived of essential treatment, potentially causing disruption or lack of availability during emergencies. Intern physicians often seek staff guidance when faced with uncertainties in their work, and their burnout is influenced by interactions with colleagues and medical staff. Despite the challenges of these interactions, it is important to note that tertiary hospitals provide a diverse range of equipment and resources to support comprehensive medical care services [43]. However, interns often avoid seeking consultation due to fear of humiliation and criticism from specialists, which not only discourages seeking help from specialists but also from their peers [44,45]. They also avoid the risk of toxic environments and open reprimands from staff [46]. This situation, which involves a complex interaction between medical necessity and interpersonal dynamics, significantly affects the clinical decision-making [47].

Violence, bullying, harassment, and abuse are prevalent issues within the medical education society [44,45], and their occurrence depends largely on the individual involved and the institutions [48]. Interns mentioned that some hospitals often report medical errors through social media or large group platforms and sometimes communicate the error directly to patients and families. This abuse, including verbal, physical, and sexual harassment, underscores the need for mutual respect and respect for humanity in this environment [49,50]. The Thai Institute of Medical Education advocates for SAFE medical school principals to create secure environments for students, including saying “no” to sexual harassment, avoiding bullying, providing appropriate feedback, and encouraging equity [51]. Importantly, hospitals hosting intern physicians should adhere to these guidelines to ensure a supportive and safe learning environment.

The COVID-19 pandemic has led to proposed policy changes aimed at optimizing clinical practices and hospital management for intern physicians in preparation for future pandemics (Figure S1). To enhance patient care and mitigate burnout among intern physicians, hospitals should refine clinical training programs and workforce allocation strategies by addressing workload distribution and accounting for hidden populations within the area [52,53]. Ensuring an appropriate physician-to-population ratio in relation to actual workload demands is crucial for maintaining healthcare efficiency. In Thailand, the employment law governing working hour restrictions does not extend to physicians. The Thai Medical Council has recommended working hour limitations for intern physicians, urging hospitals to comply. However, non-compliance carries no legal penalties. Rather, this serves as an evaluative mechanism to assess hospitals’ ability to effectively train intern physicians in the subsequent year [54].

Additionally, hospitals should establish well-structured workflow processes for intern physicians during clinical training, implementing comprehensive orientation programs to provide a clear understanding of their roles and responsibilities. Such orientation initiatives should encompass a detailed overview of annual workload expectations, specific duties for each rotation, the number of overtime shifts per ward, and transparent guidelines for consultant referrals. These measures can help minimize conflicts and misunderstandings while fostering a more collaborative working environment. Additionally, the organization of targeted workshops is essential to equip intern physicians with critical skills and protocols necessary for their duties. These workshops should include training in Advanced Cardiovascular Life Support (ACLS), essential procedures for managing life-threatening conditions—such as endotracheal intubation and intercostal drainage (ICD) insertion—and strategies for effective communication. Such initiatives are vital for enhancing teamwork, efficiency, and overall clinical preparedness [55,56].

Hospitals should regularly monitor responsiveness to complaints, address medical errors as systemic issues, foster effective problem-solving, and improve the quality of administration, particularly with regard to Hospital Accreditation (HA) [57]. Intern physicians have proposed an anonymous feedback system to address issues of colleague humiliation and violence. This would facilitate open communication without fear of reprisal, enabling intern physicians to express their emotions, share problem-solving strategies, and foster solidarity, factors that may otherwise be hindered by concerns related to training evaluations. The proposal also emphasizes the importance of implementing stringent measures to prevent sexual harassment [58]. Additionally, strengthening mental support is crucial for physicians’ well-being, ensuring they can provide high-quality care and maintain a healthy medical workforce. Physicians often work in high-pressure environments, making quick decisions, managing complex cases, and coping with patient outcomes [27,59]. This constant stress can lead to emotional exhaustion, fatigue, and difficulty maintaining empathy and engagement with patients. Addressing this emotional burden is essential for a healthy and effective medical workforce [8,13]. Future research should explore intern physician management at the policy level through interviews with key stakeholders, including the Medical Council and the Ministry of Public Health (MOPH), as well as supervisors and interns across diverse health regions to address regional differences.

The approaches employed ensure that intern physicians are well-informed about previous challenges, thereby promoting transparency, accountability, and proactive problem-solving. These approaches also cultivate a culture of mutual respect between staff and intern physicians, maintaining positive interpersonal relationships within hospital settings. The generation gap among physicians, defined by differing perspectives and behaviors across age groups, has become increasingly prominent in large hospitals, fostering collaboration across generations [52]. Modern physicians tend to be assertive and openly express their opinions, leading hospital managers to facilitate connections between intern physicians and staff members of similar ages in order to enhance communication and teamwork [52]. Physicians need to understand the context to avoid immediate responses to feedback and recognize systemic issues that may require time for adjustment and higher-level problem management, potentially extending beyond a physician’s one-year tenure. Effective coping mechanisms, including prioritizing physical well-being, establishing clear boundaries, maintaining professional passion, fostering realistic expectations, and cultivating a supportive environment, have been shown to mitigate burnout [60]. The development of resilience skills emerges as a particularly effective strategy for intern physicians, as it enhances psychological resilience and functions as a critical protective factor against burnout. Additionally, effective communication skills are crucial for the PCT to work cohesively and provide optimal patient care [61]. Newly graduated physicians must prepare for challenges both individually and within the organization by adapting to new environments and proactively anticipating potential difficulties throughout their professional journey [62,63]. Notably, developing resilience skills serves as an effective strategy to mitigate burnout, offering psychological immunity and enhancing their ability to cope with workplace demands [64].

The COVID-19 pandemic significantly impacted physicians worldwide, with intern physicians playing crucial roles in patient care under challenging conditions. Limited research exists on their experiences, particularly in resource-constrained settings. Key findings emphasize the importance of effective workforce management, streamlined workflows, sufficient resources, interpersonal skill development, and mental health support to enhance learning and performance. These insights provide hospital managers with guidance to develop comprehensive support systems for intern physicians. However, this study has several limitations. First, the small sample size of 19 participants may limit the generalizability of the findings, particularly when applied to diverse healthcare settings. Healthcare variability from differences in healthcare structures, resources, and pandemic-related challenges across regions and institutions mean that the experiences captured in this study may not fully represent those of all healthcare workers. Second, the use of purposive sampling introduces the potential for selection bias, though the study did include participants from diverse geographic regions, hospital types, and genders. Third, online interviews, which were necessary due to COVID-19 restrictions, also limited the ability to observe non-verbal cues, potentially affecting the deep contextual interpretation. Further studies should aim to involve larger, more diverse samples across various cultural contexts during health crises to enhance the robustness and applicability of the findings.

## 5. Conclusions

Intern physicians in Thailand who experienced burnout during the COVID-19 pandemic reported facing emotional suffering and chronic stress due to the changes in their work environments. These changes included extreme workloads, complicated medical procedures, and challenging working conditions and management practices. Interns also described a lack of working support, instances of humiliation by colleagues, insufficient empathy from staff, and exposure to shame-driven learning. The findings revealed that some hospitals reported medical errors on social media or in large group settings, and interns faced verbal and physical abuse, as well as sexual harassment from staff, contributing to their dehumanization and burnout. As proposed solutions, interns suggested changes in the policies of clinical practices and hospital management during future pandemics. These include improved workforce allocation strategies, clear workflow processes, fostering a culture of mutual respect between staff and interns, implementing an anonymous feedback system, and providing mental health support. Additionally, developing interpersonal skills, such as open communication and self-development, before graduation is essential for new physicians. Policy recommendations to prevent burnout among intern physicians include increasing effective clinical practice management, increasing academic support, implementing comprehensive orientation programs, fostering effective communication, enhancing teamwork assistance, managing emotional stress, and transforming organizational culture to prioritize and value physicians during their internships. Further research should examine the effectiveness of these proposed solutions in mitigating burnout among intern physicians and other specialists, and the scope of the study should be expanded to include other countries, particularly those in Western cultural contexts.

## Figures and Tables

**Table 1:** Demographics and workplace information for the participants.

Participant Number (P)	Gender	Location/ Resident Training Status	Affiliated Hospital	Hospital Bed Size
1	Female	Central/Not medical school	MOPH	606
2	Male	Central/Not medical school	MOPH	316
3	Female	Eastern/Not medical school	MOPH	312
4	Male	Southern/Not medical school	MOPH	314
5	Female	Northeast/Not medical school	MOPH	820
6	Female	Northeast/Not medical school	MOPH	974
7	Female	Northeast/Not medical school	MOPH	565
8	Male	Central/Not medical school	Autonomous hospital	323
9	Female	Southern/Medical school	MHESI	863
10	Female	Northern/Medical school	MOPH	743
11	Female	Northern/Medical school	MHESI	1400
12	Male	Northeast/Not medical school	MOD	420
13	Female	Northeast/Not medical school	MOPH	388
14	Male	Northern/Not medical school	MOPH	411
15	Female	Central/Not medical school	MOPH	316
16	Female	Northeast/Not medical school	MOPH	301
17	Female	Northeast/Not medical school	MOPH	895
18	Male	Northeast/Not medical school	MOPH	832
19	Female	Southern/Medical school	MHESI	853

Abbreviations: MOPH, Ministry of Public Health; MHESI, Ministry of Higher Education, Science, Research and Innovation; MOD, Ministry of Defense.

**Table 2:** Interview open-ended questions for the participants.

Questions	
1.	Which hospital are you currently working at? How many beds does the hospital have, and what is its overall service capacity?
2.	Could you describe the nature of your work over the past year? Please include information about the locations of your rotations, the number of shifts you worked per week, and details regarding your income.
3.	Please explain your experiences and feelings while providing care to patients during the COVID-19 pandemic, particularly if you felt pressured, stressed, or burned out.
4.	What traumatic events (contributing to your feelings) did you go through when this duration was going on?
5.	How did you address the work-related issues that led to your burnout, and what were the results?
6.	How did this situation affect your working relationships, for example with colleagues and staff?
7.	How did the situation affect your learning practice?
8.	What were the main challenges in your professional and personal life in this situation?
9.	What potential solutions would you give to hospital administrators and policymakers to reduce burnout for intern physicians?
10.	What suggestion would you give to incoming intern physicians to prevent burnout?

**Table 3:** Two parts, themes, and their subthemes extracted from data analysis.

Themes	Subthemes
Part 1: Experiencing burnout during intern physician’s life	
Emotional suffering and stress	Exhaustion and pressure in daily responsibilities Unhappy: lifestyle and imbalance in work and play
Engaging in high-intensity workplace	Extreme workload while lacking colleague support Facing complicated tasks beyond responsibilities Highly demanding but unable to control
Hostile work environments	Insufficient work support Being humiliated by colleagues Interns’ voices: remaining unresolved
	Shame-driven learning
Part 2: Suggesting potential solutions for clinical practices and hospital management	
Changes in policies for clinical practices	Effective workforce and shift allocation Strong workflow process
Effective hospital management	Promotion of a culture of mutual respect between staff and interns Anonymous workplace reporting Strengthening mental health support
Building interpersonal skills	Effective communication Self-development before graduation

## Data Availability

The data presented in this study are available from the corresponding author on reasonable request.

## Supplementary Materials

The following supporting information can be downloaded at: https://www.mdpi.com/article/10.3390/healthcare13030335/s1, Figure S1: Experiencing burnout among first-year intern physicians and proposed solutions for policy changes, effective hospital management and interpersonal skills for intern physicians during the next pandemic.
