# Supplementary material for: Examining Burnout Among Intern Physicians During the COVID-19 Pandemic: Insights and Solutions from Qualitative Research
Source: Healthcare (Basel). 2025 Feb 6;13(3):335. doi: 10.3390/healthcare13030335 (PMC11816800; doi:10.3390/healthcare13030335)
Supplement: Supplementary file 1 [file healthcare-13-00335-s001.zip › healthcare-3419751-supplementary.pdf]

Experiencing burnout of first-year intern physicians in governmental hospitals during the COVID-19 pandemic

Emotional suffering and stress

Exhaustion and pressure in daily responsibilities

Unhappy: lifestyle and imbalance in work and play

Engaging in high-intensity workplace

Extreme workload while lacking colleague support

Facing complicated tasks beyond responsibilities

Highly demanding but unable to control

Hostile work environments

Insufficient work support

Being humiliated by colleagues

Interns' voices: remaining unresolved

Shame-driven learning

Proposed solutions for policy changes, effective hospital management, and interpersonal skills

Changes in policies for clinical practices

Effective workforce and shift allocation

Strong workflow process

Effective hospital management

Promotion of a culture of mutual respect between staff and interns

Anonymous workplace reporting

Strengthening mental support

Building interpersonal skills

Effective communication

Self-development before graduation

Figure S1. Experiencing burnout among first-year intern physicians and proposed solutions for policy changes, effective hospital management and interpersonal skills for intern physicians during the next pandemic.
